# A Novel Peer-to-Peer Coaching Program to Support Digital Mental Health: Design and Implementation

**DOI:** 10.2196/32430

**Published:** 2022-01-26

**Authors:** Benjamin M Rosenberg, Tamar Kodish, Zachary D Cohen, Elizabeth Gong-Guy, Michelle G Craske

**Affiliations:** 1 Department of Psychology University of California, Los Angeles Los Angeles, CA United States; 2 Semel Institute for Neuroscience and Human Behavior University of California, Los Angeles Los Angeles, CA United States; 3 Department of Psychiatry and Biobehavioral Sciences University of California, Los Angeles Los Angeles, CA United States

**Keywords:** peer support, digital mental health, university students, college students, training and supervision, scalable psychological interventions

## Abstract

Many individuals in need of mental health services do not currently receive care. Scalable programs are needed to reduce the burden of mental illness among those without access to existing providers. Digital interventions present an avenue for increasing the reach of mental health services. These interventions often rely on paraprofessionals, or coaches, to support the treatment. Although existing programs hold immense promise, providers must ensure that treatments are delivered with high fidelity and adherence to the treatment model. In this paper, we first highlight the tension between the scalability and fidelity of mental health services. We then describe the design and implementation of a peer-to-peer coach training program to support a digital mental health intervention for undergraduate students within a university setting. We specifically note strategies for emphasizing fidelity within our scalable framework, including principles of learning theory and competency-based supervision. Finally, we discuss future applications of this work, including the potential adaptability of our model for use within other contexts.

## Mental Health: A Global Crisis

### Background

Mental illness is a pressing and growing global public health crisis with enormous societal costs [1]. Between 1990 and 2017, the number of cases of depression worldwide grew from 172 to 258 million [2]. Unfortunately, the majority of people in need of treatment do not receive care, due to a multitude of factors that reduce availability and accessibility of mental health services [3]. For instance, worldwide, shortages in trained professionals and resources allocated for mental health care limit access to treatment [4]. Although evidence-based treatments (EBTs) exist for mental health disorders, there is a major lag in translation of these treatments from laboratories to the real world [5]. Projections indicate that significant shortages of mental health practitioners will continue throughout the next decade, underscoring the need for innovative and scalable solutions to deliver EBTs [6,7].

One widely studied scalable approach, used most prominently in low-resource contexts, is for paraprofessionals to provide or support the delivery of scalable mental health services [8,9]. In this paper, we use the term “paraprofessionals” to refer to nonspecialists without formal mental health credentials who are trained to provide or support low-intensity mental health services in community settings. Under this umbrella, we include individuals who have been described using a variety of terms, such as “coaches,” “lay providers,” “community health workers,” and “peer specialists” [10-12]. Although paraprofessional support models represent a clear pathway to increasing access to care, little is known about the training, quality of care delivery, and sustainability of these models.

Digital mental health innovations via phone, computers, and other electronic devices offer another pathway for increasing access to care [13]. Digital mental health interventions hold particular promise for individuals who face obstacles to traditional, face-to-face mental health services, such as stigma, financial difficulties, time constraints, and location of services [14]. Although user uptake, engagement, and dropout have been problematic for digital mental health interventions [15], especially in routine clinical care settings [16], these problems can be addressed via human support [17-19].

Accordingly, mental health care models that combine paraprofessional workforces and digital mental health innovations have unique potential to expand the reach of and engagement with high-quality EBTs. One key consideration in efforts to design and implement paraprofessional-supported digital mental health interventions involves balancing *scalability*, to maximize intervention reach, with *fidelity*, to optimize quality and standards of treatment delivery. Scalability can be defined as “the capacity of an intervention to be applied in a way that reaches a large number of people” [6]. Fidelity encompasses both adherence (ie, Was the intervention delivered as intended?) and competence (ie, How skillfully was the intervention delivered?) [20] to ensure that patients receive efficacious treatment that leads to improved mental health outcomes [21].

### Study Aim

The purpose of this paper is to demonstrate 1 way of designing a coaching program that maintains a focus on the fidelity and delivery of high-quality EBTs, while preserving key strengths of paraprofessional models of care, including scalability. Our program was developed to support the delivery of a digital mental health intervention [22] on college campuses, where rates of mental health problems are rapidly growing [23]. Given the current state of the literature, we first describe gaps in our knowledge about the fidelity of treatment delivery within existing paraprofessional programs, such as peer-to-peer support programs. Next, we highlight how pairing digital mental health innovations with paraprofessional support can increase the fidelity and scalability of mental health treatment. Third, we describe our approach to the design and implementation of a peer-to-peer training program, emphasizing potential avenues for optimizing learning processes to enhance the fidelity of treatment delivery.

## Paraprofessional Mental Health Delivery Paradigms

### Scalability and Fidelity

Paraprofessional models have gained widespread attention and support as scalable models of mental health service delivery with great potential to address unmet needs for care [8,24]. Evidence suggests that mental health interventions can be feasibly, acceptably, and effectively delivered by paraprofessionals in low-resource settings [13]. Paraprofessional training programs have the added benefit of increasing the clinical workforce, as these individuals often move on to receive advanced training in the clinical field after serving as paraprofessionals [25].

Fidelity-monitoring practices have the capacity to increase therapist accountability in service of promoting treatment adherence and competence [26]. Indeed, greater therapist competence has been associated with superior treatment outcomes [27]. However, numerous challenges with fidelity monitoring have been identified in the context of paraprofessional service delivery [8,28], such that existing paraprofessional care programs have focused primarily on scalability needs, with less attention given to fidelity of service delivery [29]. Given pressing demands to rapidly reach millions of underserved individuals in need, even paraprofessional interventions that are supported by research and contain evidence-based strategies often lack consistent fidelity-monitoring and quality assurance procedures. For instance, only 38% of studies in a review of community health worker–delivered interventions described procedures for fidelity monitoring, and among those that did report a monitoring procedure, the review noted significant variability in levels, methods, and assessment tools for fidelity measurement [8].

The financial and human resources needed to support fidelity monitoring in real-world contexts are often not available, limiting the external validity of many fidelity-monitoring strategies typically used in clinical trials [30]. Even when fidelity and quality assurance checks are integrated into training and supervision within paraprofessional models, sustained fidelity monitoring is often restricted due to limited supervision and insufficient resources to ensure continued quality assurance [28,30]. Paraprofessional programs delivered with less fidelity monitoring are thought to reduce intervention efficacy [27] and may discourage participants from future engagement in treatment. Randomized control trials have shown that with adequate training and ongoing supervision, paraprofessionals have the capacity to deliver interventions with similar levels of fidelity compared to mental health professionals [31,32]. However, less is known about how to design and implement high-fidelity training programs in more scalable contexts. Qualitative research suggests that lay health workers involved in mental health service delivery state a desire for more robust supervision. Yet, training and supervision best practices have not been established to date [33]. The limited research describing training and supervision procedures in paraprofessional delivery paradigms underscores the need for innovative solutions that have dual goals of sustaining potential for scalability, while also ensuring the fidelity of intervention delivery.

### Pairing Technological Innovation With Paraprofessional Support to Enhance Fidelity and Scalability

Digital therapies hold significant promise for addressing problems with fidelity and bridging gaps in care access within wide-scale implementation efforts [27,30]. In particular, these approaches offer 1 way to support treatment delivery, paraprofessional training, and supervision, while minimizing human error or therapist drift, a common phenomenon in manualized treatment protocols [34]. Although humans often play a smaller role within digital therapy models relative to traditional face-to-face therapy, human support or coaching has been shown to augment the efficacy of digital interventions [35]. This is particularly important, given the many challenges and barriers associated with implementation of digital therapies, including limited engagement, poor rates of retention, lack of personalization, and significant cognitive load [15,36]. The involvement of human support increases intervention flexibility and acceptability by calibrating the fit between digital tools and users’ lived experiences, thereby boosting user engagement and retention [18,37]. Lattie et al [38] provide recommendations for the development of text-based coaching protocols (eg, [39]) to support digital mental health interventions and ensure high-fidelity treatment delivery. Thus, pairing paraprofessional coach support with digital therapies has several notable advantages that attend to the need for scalable innovations, while simultaneously emphasizing fidelity.

### Peer-to-Peer Support

One consideration in designing paraprofessional models is who should be trained to provide, or support the delivery of, mental health interventions. A prominent model focuses on training of peer-to-peer specialists, or peer coaches [40]. Peer coaching models have been used to provide services or support to individuals with whom coaches share communities, identities, or lived experiences, with the goal of enhancing accessibility, engagement, and scalability of interventions [41]. In doing so, these models have the potential to overcome obstacles to care, such as lack of trust, stigma, and cultural and linguistic barriers (although the significance of peers’ own lived experiences is yet to be determined). One common example is peer recovery and support for individuals with substance use disorders [42], where a peer’s own experience and personal knowledge is harnessed to support individuals in starting and maintaining the recovery process [43-45]. Key legislation is paving the way to expand peer specialist programs to address a variety of population mental health needs, such as the 2020 California Senate Bill SB-803: Mental Health Services: Peer Support Specialist Certification.

Yet, a major barrier to broader implementation of peer support is the mixed empirical support for these models [46-49]. There is some evidence to suggest more positive effects from formal, structured peer support (eg, [50-53]) than informal support (eg, online chat forums) [54,55]. Nonetheless, the findings are inconsistent even within structured peer support interventions (eg, [56]). Methodological inconsistencies may partly explain the disparate findings [42,56], and 1 major example is training and quality assurance. Standardized procedures for peer training, certification, and fidelity monitoring are not well described in the literature [47,56]. Well-defined and replicable methods for training and quality assurance procedures are sorely needed.

## Design of Coach Training Programs

### Overview

**Figure 1:**
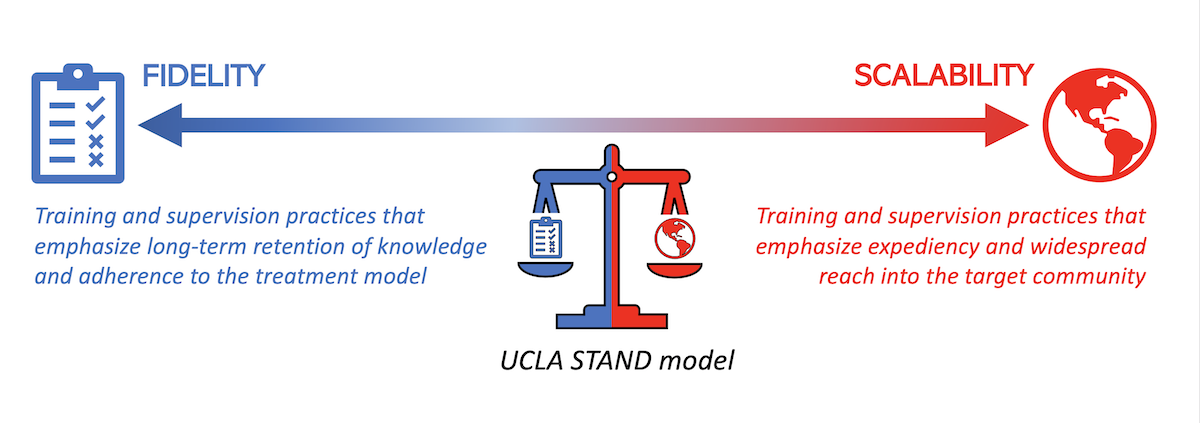
Navigating scalability and fidelity in mental health coaching programs. STAND: Screening and Treatment for Anxiety and Depression; UCLA: University of California, Los Angeles.

In 2015, the University of California, Los Angeles (UCLA) launched a campus-wide research initiative, the Depression Grand Challenge (DGC), with the goal of cutting the burden of depression in half by 2050. The DGC comprises a number of studies that seek to uncover mechanisms underlying depression and to develop novel treatments and innovative approaches to treatment implementation. To begin tackling this problem at UCLA, the DGC launched the Screening and Treatment for Anxiety and Depression (STAND) program for UCLA students in fall 2017 (Figure 1). The STAND program provides all UCLA students with free mental health screening and tiered care, including digital cognitive-behavioral therapy (CBT) with certified peer coach support for students experiencing mild-to-moderate symptoms of depression and mild-to-severe symptoms of anxiety, as well as in-person psychotherapy and pharmacotherapy for students experiencing severe symptoms of depression. Students who enroll in the digital CBT arm are offered coaching from certified peers, provided via 30-minute weekly coaching sessions in which they review and troubleshoot the application of module content and skills.

STAND Digital Therapy is a modular program that combines interventions for depression, sleep, panic/agoraphobia, social anxiety, worry (generalized anxiety disorder), and trauma (posttraumatic stress disorder), drawing upon existing evidence-based programs [57-66]. There are 13 available packages that cover all principal disorders and critical patterns of comorbidity (eg, depression + sleep, trauma + depression) and comprise 6-8 modules, depending on the number of disorders targeted. Individuals are assessed at baseline on an adaptive battery of disorder-specific, self-report questionnaires that guide the package selection process [22]. The personalized packages are built to maximize engagement and interactivity and with a strong focus on diversity and inclusion. The modules are transdiagnostic and skill focused, involving psychoeducation, in session exercises, and between-session practice of techniques, including behavioral activation, cognitive restructuring, self-compassion, and exposure (eg, in vivo, interoceptive, imaginal).

Fitting within this model, the initial development of our coach training program specifically targets UCLA undergraduate students as both coaches and recipients of the intervention, consistent with the peer support models described before. Enrollment as a coach trainee does not rely on any prerequisite coursework, history of service provision, or experience of personal mental health concerns or psychotherapy. Training and supervision of coaches are provided by graduate students in the clinical psychology doctoral program at UCLA for all stages of coach training. Graduate supervisors attend group supervision-of-supervision with a licensed clinical psychologist (author EGG).

### Program Description

In our program, coach training occurs in weekly sessions, wherein trainees review digital CBT content, engage in didactic instruction of coaching materials, and complete role-play exercises focusing on basic interpersonal process skills. Coaches move through 4 primary phases of training: (1) beginner, (2) intermediate, (3) advanced, and (4) certified. Weekly training consists of a 2-hour training session as well as 2 hours of assignments completed between training sessions. Each level of training is completed over 1 academic quarter (10 weeks), at which point trainees are advanced to the subsequent level of training based on supervisor evaluations.

#### Beginner-Level Training

The goals of the beginner phase of training are to (1) introduce coaches to digital CBT content and increase knowledge of the intervention and (2) provide early practice with interpersonal process skills to initiate the process of translating declarative knowledge during coaching delivery. In service of these aims, beginner-level trainees enroll as users of the digital CBT and advance through the digital CBT content themselves, completing homework exercises associated with the program and reading foundational material on cornerstone CBT topics between didactic training sessions. In addition, beginner-level trainees are introduced to 6 core interpersonal process skills that are routinely assessed to monitor coaching effectiveness throughout the coach training program: (1) authenticity, (2) nonverbal skills, (3) open-ended questioning, (4) reflecting emotions, (5) content summaries, and (6) collaborative inquiry [67-69]. These process skills, in addition to sustained knowledge of the digital CBT content, provide the foundation for advancement throughout the coach training program.

Beginner-level trainees participate in (1) didactics regarding digital CBT content and interpersonal process skills, (2) discussions regarding other cornerstone topics (eg, mindfulness, cultural humility, trauma-informed care, ethics), and (3) role-play exercises to begin practicing application of the 6 core interpersonal process skills. Beginner trainees also attend sessions with advanced trainees, in which they serve as mock or practice participants for advanced trainees who are coaching full mock sessions (described in detail in the Advanced-Level Training section). Role-play exercises are recorded or observed live by supervisors, who provide oral and written feedback, as well as numerical ratings on each interpersonal process skill (eg, scale from 1 to 10 with behavioral anchors; see Multimedia Appendix 1). These evaluations provide benchmarks for certification and highlight areas of growth as trainees progress toward certification throughout the program.

#### Intermediate-Level Training

As trainees progress into the intermediate stage of the program, the primary goals are to provide trainees with intensive practice, (1) translating knowledge into coaching delivery and (2) applying interpersonal process skills to support engagement with digital CBT content. During these sessions, trainees participate in (1) brief digital CBT module content review, (2) intensive role-play exercises applying core process skills, and (3) introduction to protocols for managing advanced clinical issues (eg, suicidality, homicidality, abuse).

To continue supporting trainee development of interpersonal process skills and digital CBT content knowledge, trainees are continually rated on their process skills throughout intensive role-plays. Each week, supervisors review trainees’ intensive role-play segments and provide trainees with written feedback and numerical ratings on core interpersonal process skills. In addition, group supervision sessions incorporate oral feedback from supervisors and peer coaches, including in vivo corrective feedback during role-play exercises.

#### Advanced-Level Training

Once trainees reach the advanced stage, the main goal is for trainees to achieve certification to serve as coaches for participants. This is accomplished by demonstrating (1) competency across all 6 core interpersonal process skills and (2) continued knowledge of digital CBT content. Advanced trainees conduct practice coaching sessions (ie, full 30 minutes) with beginner trainees as mock participants. In addition to these practice sessions, advanced trainees attend a weekly supervision group consisting of intensive role-play exercises, with role-play targets focused on digital CBT content, interpersonal process skills, and management of advanced clinical issues (eg, suicidality, homicidality, abuse, sexual assault, self-disclosure).

To support advanced coaches in progressing toward certification, advanced-level trainees receive written and numerical ratings on their full 30-minute practice coaching sessions. These ratings are used to certify trainees on competency across all process skills. Next, trainees achieve certification on digital CBT content by passing quizzes, which ensures knowledge of the intervention and promotes continued fidelity to the treatment model.

#### Coach Certification

Following successful advancement through the prior 3 stages of the program, trainees are certified to support the digital CBT with continued supervision. Certified trainees who are engaged in coaching continue to attend weekly supervision groups in which they discuss coaching sessions with their supervisor and peers. To ensure continued fidelity to coaching standards, supervisors review video recordings of each coaching session and rate the coaches’ application of process skills according to the behavioral rating scale described before. Video review further enables supervisors to use didactics and role-play exercises in response to common challenges or to address drift from the coaching protocol. Certified trainees additionally provide feedback to the supervision team to inform potential future iterations of the coaching program.

### Strategies for Monitoring and Enhancing Fidelity

#### Learning Theory

Increased attention to trainee learning processes within mental health provider training and supervision procedures has potential to increase fidelity to EBTs [70]. One way to enhance paraprofessional mental health service delivery, therefore, is to design training programs leveraging insights from learning theory and the use of specific pedagogical strategies (see Table 1 for examples) shown to improve knowledge building, skill acquisition, and long-term retention across domains such as learning a new language, mathematics, and sports [71-73]. Although these strategies may reduce performance in the short term (ie, during initial acquisition of skills or knowledge), research has consistently shown superior long-term retention and retrieval of learning [72,74].

**Table 1:** Pedagogical strategies and examples.

Principle	Definition	Example
Varying context of learning	Incorporating contextual variability (eg, physical location, types of teaching strategies) into teaching and learning	Compared with individuals who repeatedly study in 1 setting, individuals who study in a variety of physical settings have been shown to perform better on subsequent examinations in a new setting [75].
Spaced instruction	Spacing out instruction of a single topic over a period, as opposed to solely providing instruction about a topic in 1 learning event	Although cramming for an exam may be a useful strategy for performing well in the short term (eg, on a quiz), spacing the presentation of materials over a longer period has been shown to support performance in the long term (eg, on a final examination).
Interleaved instruction	Interleaving instruction of different topics within a common learning event (eg, covering multiple concepts within a single class)	Interleaving questions that assess knowledge of multiple concepts (eg, geometric equations for angles and lines intermixed) has been shown to improve student learning compared with blocking of concepts (eg, equations for angles, then lines) [76].
Retrieval practices/ examinations	Formal assessment of knowledge (eg, tests, assessments, exams)	Individuals who make incorrect guesses have been shown to benefit from these early mistakes during learning compared with individuals who are provided with the correct answers from the beginning of training [77].

#### Learning Theory: Applied

From the outset of coach training, we have applied core principles of learning theory to guide the instruction of digital CBT content and process skills. For example, *variability of learning contexts* is applied through (1) independent trainee review of digital CBT content (outside of sessions), (2) didactic training (during sessions), (3) role-play exercises (conducted in small groups), and (4) participation in mock sessions (observed by the entire supervision group). Likewise, applying the principle of *spaced instruction*, digital CBT content and interpersonal process skills are introduced and revisited at multiple timepoints within and across training levels. *Interleaved instruction* is similarly used to promote initial learning of digital CBT content and process skills simultaneously (eg, a single training session alternates between CBT and process skill content, and likewise combines the 2 domains, rather than blocking 1 instruction topic at a time). Furthermore, *retrieval practices* assess digital CBT knowledge throughout all stages of trainee development to support long-term retention of learning (eg, during the advanced stage of coach training, the process of obtaining certification involves trainees repeatedly completing mock coaching sessions with corrective feedback).

Following certification, ongoing fidelity-monitoring practices include (1) completion of a self-evaluation coaching checklist following all coaching sessions, (2) discussion of coach adherence to the digital CBT module during supervision, and (3) continued completion of mock coaching sessions during supervision with peer-to-peer and supervisor feedback.

#### Competency-Based Supervision

Following the acquisition of new knowledge and skills, competency-based supervision techniques can provide trainees with a pathway for transforming declarative knowledge into procedural knowledge [78-81]. Prior studies support the notion that competency-based supervision can increase effective CBT knowledge and acquisition [82]. Accordingly, the present coach training program integrates experiential learning and competency-based supervision strategies to support sustained fidelity to the treatment. For example, our program uses supervision practices that integrate a variety of experiential learning techniques (eg, skill modeling, role-plays, and corrective feedback), which have been shown to increase provider fidelity to EBTs [70]. Likewise, the program continuously assesses and monitors trainee development with clearly articulated, behaviorally anchored feedback [81].

## Discussion

### Principal Findings

In this paper, we outlined 1 example of a scalable peer-to-peer mental health paraprofessional training and supervision program. Although many models of paraprofessional support have been described and tested previously, high demand and minimal resources have often corresponded with a reduced focus on fidelity monitoring and quality assurance [8]. Lack of standardized methods for paraprofessional training and supervision may have contributed to the disparate empirical support for paraprofessional, and specifically peer paraprofessional, models. Here we described a standardized and replicable model of training and supervision suitable for evaluation.

### Strengths

We believe this model has several notable strengths. Of note, our program focuses explicitly on fidelity, while also attending to the need for scalable care. As illustrated, the focus on fidelity is integrated into the program in 2 primary ways: digital technology as the primary agent for CBT content delivery [83] and continuous, standardized procedures for fidelity monitoring of coaches who support digital CBT provision. In addition, our training and supervision program is grounded in key findings from the learning theory literature, aligned with data suggesting that optimized learning can serve as a pathway to higher fidelity of treatment delivery [70,78]. The integration of learning theory as a mechanism for enhancing fidelity is aligned with existing lay health worker training frameworks that focus on augmenting initial one-off training with on-the-job direct supervision, coaching, and feedback systems [28]. We believe that paraprofessional models anchored in learning theory principles have the greatest potential to improve quality of care.

Another strength is that our program is designed to be malleable and can be adapted in various ways based on implementation context factors. Along with fidelity, program flexibility is well established as a key ingredient to successful implementation of interventions in numerous settings [84,85]. Implementation science frameworks have frequently cited the importance of balancing both fidelity and flexibility in delivery of EBTs, and this concept has also been established as essential in lay health worker models [28]. Our program was designed with flexibility within fidelity as a key guiding principle. It contains both *core components*, defined within the Consolidated Framework for Implementation Research (CFIR) as the “essential and indispensable elements” of the program, and the *adaptable periphery*, defined as the aspects of the program that can be modified and varied from site to site [86,87]. Included in our program’s core components are (1) anchoring in principles of learning theory described before, (2) training on 6 core clinical process skills, and (3) training on digital CBT content. The adaptable periphery, however, depends on the structures, systems, and contexts involved with program implementation. In the process of designing adaptations, community stakeholder partnership and input are essential [88]. Although many adaptation frameworks have focused on adaptations to the intervention itself, stakeholders can also be used to consider adaptations to the implementation context.

In our program, we have identified several components of the adaptable periphery that have been tailored for various implementation contexts, with community partnership. For instance, although this paper describes implementation at 1 university, we are currently piloting coach training and supervision for the launch of STAND digital CBT in numerous other types of community settings, including local community colleges and health care systems. In partnership with community stakeholders, 1 example of a component in the adaptable periphery that we have modified to meet the needs of a new implementation site is the length of training time, which has been shortened to accommodate local resources. This has been accomplished by combining components of beginner and intermediate levels of training and including additional review and feedback of recorded role-plays outside of sessions to accelerate learning and growth. In another example of adaptation, we have worked with various sites to situate and design our coaching risk protocols (eg, suicide risk, abuse) within the contexts of existing resources, infrastructure, and referrals. Another example of adaptation has been to integrate specific training on trauma-informed care strategies to support implementation of this program in communities with higher trauma prevalence rates. Cultural considerations are also essential, particularly in planning implementation of coach training programs in diverse settings such as ours. Working in partnership with community stakeholders to co-design cultural adaptations can lead to improved program acceptability and community engagement. Although we have made and discussed modifications within the adaptable periphery based on the unique implementation and contextual factors within various environments, the same guiding principles described in this paper serve as the foundational core components across settings.

A final strength of our program is that it is intended not only to train students to serve as coaches to their peers but also to provide critical CBT skills to trainees themselves. Many coaches in our program anecdotally report that their experience throughout training has taught them invaluable interpersonal and cognitive-behavioral skills. In the broader literature, paraprofessionals describe feeling that their training experiences were associated with personal development and growth and increases in knowledge, self-confidence, and skill use [33]. In the context of our program, formal measurement of mental health benefits conferred by coaches in our program is needed.

### Limitations

Several key limitations of our program should also be noted. First, because this program is situated within the scope of a large research initiative, ongoing funding has been available to sustain coach training and supervision. Beyond the realm of research, efforts to provide continuous funding for paraprofessional support programs in routine care settings are critical. In the initial iteration of our program, coaches have served as volunteers, engaged in all program elements as an additional responsibility outside of their other obligations. Data suggest that among volunteer staff supporting digital interventions, administrative issues, such as time constraints, may contribute to barriers to training completion and attrition [89]. Additional funding that encompasses financial payment or other incentives for peer coaches may represent 1 solution to address this obstacle. One model that is currently being tested as a component of our program’s adaptable periphery is paying coaches as university employees. Alternative methods of expanding and sustaining funding and resources are worthy of exploration.

Second, although we maintain a focus on fidelity in our program, the primary objective of our peer-to-peer program is to serve as a scalable model of care in real practice settings. Thus, given the resource constraints of real-world implementation contexts, we have designed our fidelity-monitoring procedures to minimize supervisor and trainee burden. However, in doing so, we recognize limitations in our capacity to optimally monitor fidelity, and acknowledge that fidelity is not monitored to the same degree in our program compared to standard clinical trials (eg, [90]).

Third, to maximize scalability of the program, coaching is provided virtually using videoconferencing. Prior research has raised the possibility that compared with self-administered or fully automatized options, digital mental health interventions may be most effective for adolescents and young adults when incorporating in-person elements [91]. However, the extent to which virtual interactions with a human coach may provide a similar degree of benefit is unknown. Additional research may clarify the effectiveness of fully remote coaching and guide potential adaptations to this program.

Last, our program was initially designed for use in a specific setting (ie, a peer-to-peer program supporting college students). Additional efforts and reliance on existing implementation science and human-centered design frameworks, such as the CFIR, are needed to determine how this program and similar ones may be adapted and augmented for use in other types of settings and with new populations. A number of conceptual frameworks to adapt interventions in new contexts have been proposed, and these can be used to guide adaptation of paraprofessional support programs for new settings (eg, [92]).

### Conclusion and Future Directions

Finally, we consider future directions for this work, falling within the scope of the paraprofessional field at large. First, to meet rising rates of mental illness worldwide, expansion of paraprofessional mental health programs into new settings is critically needed. Second, funding for these programs must also encompass sufficient resources to support quality assurance in training, supervision, and treatment delivery [93], as has been the case throughout the development of the coach training program presented here. However, fidelity assurance strategies must be integrated with careful awareness of their scalability, enabling paraprofessional programs to continue expanding in reach. Third, adaptations should be designed in collaboration with community stakeholders to reduce drift from EBT protocols, while also addressing the implementation factors that drive adaptation needs [92]. Lastly, research protocols (eg, [94]) should be developed to enable empirical testing of our model, along with potential model adaptations to determine effectiveness and inform modifications to future iterations of the coach training program.

## Appendix

Multimedia Appendix 1 Rating form to evaluate interpersonal process skills.

## Abbreviations

CBT: cognitive-behavioral therapy
CFIR: Consolidated Framework for Implementation Research
DGC: Depression Grand Challenge
EBT: evidence-based treatment
STAND: Screening and Treatment for Anxiety and Depression
UCLA: University of California, Los Angeles
