# Supplementary material for: A Novel Peer-to-Peer Coaching Program to Support Digital Mental Health: Design and Implementation
Source: JMIR Ment Health. 2022 Jan 26;9(1):e32430. doi: 10.2196/32430 (PMC8829693; doi:10.2196/32430)
Supplement: Multimedia Appendix 1 [file mental_v9i1e32430_app1.pdf]

Process Skill & Module Integration Rating Anchors

Categorical Ratings	Near Absence of Demonstrated Skills/ Needs Remediation		Rarely and Inconsistently Demonstrated/ Beginner Level		Frequently Demonstrated with Occasional Lapses/ Intermediate Level		Frequently and Consistently Demonstrated / Advanced Level		Consistently and Skillfully Demonstrated / Exemplary Level	
**Numerical Ratings**	1	2	3	4	5	6	7	8	9	10
**Authenticity**	Completely preoccupied with own performance as evidenced by fidgeting, not looking at screen, long pauses, overly scripted or ridged responses<br><br>No attention toward participant		Often preoccupied with own performance as evidenced by fidgeting, not looking at the screen, long pauses, overly scripted or ridged responses<br><br>Some attention toward participant		Approximately half of attention on own performance and half attention toward participant		Little to no preoccupation with own performance<br><br>Consistent authenticity throughout session, as evidenced by focus/interest in participant, expression of caring and warmth, and present moment awareness		Little to no preoccupation with own performance<br><br>Consistent authenticity throughout session, as evidenced by focus/interest in participant, expression of caring and warmth, and present moment awareness	
**Non-verbal**	Not observed; no nodding, blank or blunted facial expression; glancing off screen, looking at notes, fidgeting		Rarely or occasionally employed non-verbal cues (e.g., occasional nods, shifts in facial expression), appeared blunted at times		Frequently demonstrated non-verbal cues through eye contact and other forms of body language (e.g., nodding, leaning into camera, smiling, etc.) to convey warmth and concern.<br><br>1-2 instances in which more nonverbal communication was indicated or nonverbal cues were incongruent with content shared by participant		Consistently employed non-verbal cues to genuinely convey empathy and concern, but most frequently engaged when speaking versus listening<br><br>Nonverbal cues were congruent with content shared by participant		Continuous and skillful use of non-verbal cues when speaking AND listening to convey genuine interest, care, and validation through natural shifts in body language/facial expressions, skillful variation in tone, and pacing of material	
**Open-Ended Questioning**	Did not ask any open-ended questions (only yes/no questions or no questions)		Rarely or occasionally asked open-ended questions, but more frequently asked closed-ended or leading questions		Frequently asked open-ended AND close-ended questions or asked closed ended questions but corrected themselves/ tried to reframe more open-endedly		Consistently asked open-ended questions that conveyed curiosity about participant's experiences, thoughts, beliefs, etc.		Consistently and effectively used open-ended questions as a natural part of conversation. Flexibly responded to participant's statements with meaningful open-ended questions that conveyed concern and genuine curiosity about participant's experience	

Process Skill & Module Integration Rating Anchors

<table>
<tr><th>Categorical Ratings</th><th colspan="2">Near Absence of Demonstrated Skills/ Needs Remediation</th><th colspan="2">Rarely and Inconsistently Demonstrated/ Beginner Level</th><th colspan="2">Frequently Demonstrated with Occasional Lapses/ Intermediate Level</th><th colspan="2">Frequently and Consistently Demonstrated / Advanced Level</th><th colspan="2">Consistently and Skillfully Demonstrated / Exemplary Level</th></tr>
<tr><th>Numerical Ratings</th><th>1</th><th>2</th><th>3</th><th>4</th><th>5</th><th>6</th><th>7</th><th>8</th><th>9</th><th>10</th></tr>
<tr><td>Reflecting Emotions</td><td colspan="2">Did not use any reflections or used invalidating reflections (e.g., pt. endorses anxiety and coach says, "oh it's not so bad")</td><td colspan="2">Occasional reflections or only used generic reflections (e.g., that sounds hard/stressful/difficult)

Delivery of validation was not paired with emotion congruent style and non-verbal cues (e.g., neutral tone when validating pain associated with loss of family member)</td><td colspan="2">Accurately identified participant's emotions in some reflections, but frequently used generic reflections

Delivery of validation was sometimes paired with emotion congruent style and non-verbal cues (e.g., softening tone, pausing, furrowing brow to reflect grief)</td><td colspan="2">Consistently identified participant's emotions and demonstrated empathy using some levels of validation

Delivery of validation was often paired with emotion congruent style and nonverbal cues (e.g., softening tone, pausing, furrowing brow to reflect grief)</td><td colspan="2">Consistently and effectively reflected participant's emotions using all levels of validations and expanding on or adding insights to convey deeper understanding

Delivery of validation was always paired with emotion congruent style and nonverbal cues</td></tr>
<tr><td>Content Summaries</td><td colspan="2">Did not use content summaries</td><td colspan="2">Occasionally used content summaries or did not accurately summarize</td><td colspan="2">Usually accurately summarized content but could have been more clear, concise, or frequent</td><td colspan="2">Consistently used accurate, clear, and concise content summaries</td><td colspan="2">Consistently and skillfully used clear, concise content summaries throughout to move the discussion forward and highlight key points</td></tr>
<tr><td>Collaborative Inquiry (Avoids Advice)</td><td colspan="2">Used direct advice and direct instructions (e.g., you should do XYZ)

Not collaborative</td><td colspan="2">Avoided direct advice; frequently used "quizzy" questions (e.g., can you tell me what each letter of SMART stands for? Yes, S is for specific) and teachy style

Not collaborative</td><td colspan="2">Avoided direct advice; inconsistent use of questions, summaries, and reflections that created opportunities for the participant to arrive at increased insight about a tool/module

Inconsistent evidence of collaboration</td><td colspan="2">Consistent use of questions, reflections, and summaries that created opportunities for the participant to arrive at increased insight about a tool/ module

Consistent evidence of collaboration (e.g., determined participant's preferences and needs in coaching session, elicited feedback and reactions throughout session)</td><td colspan="2">Continuous use of questions, reflections, and summaries that created opportunities for the participant to arrive at increased insight about a tool/ module

Continuously strong collaboration that empowered the participant as an expert in their own experience</td></tr>
</table>

Process Skill & Module Integration Rating Anchors

Categorical Ratings	Near Absence of Demonstrated Skills/ Needs Remediation		Rarely and Inconsistently Demonstrated/ Beginner Level		Frequently Demonstrated with Occasional Lapses/ Intermediate Level		Frequently and Consistently Demonstrated / Advanced Level		Consistently and Skillfully Demonstrated / Exemplary Level	
Numerical Ratings	1	2	3	4	5	6	7	8	9	10
Integration of Module Specific Content	No discussion or complete misunderstanding of module; focused on process skills with limited acknowledgement of module content		Rarely discussed module target; and/or when discussed appeared to have poor understanding of how to apply module target to pt.'s concerns; may cover multiple module tools without applying to pt.'s concerns; limited integration of process skills with module target (e.g., focused on teaching content)		Typically, clear understanding and incorporation of module tools and targets throughout session; 1-2 instances in which it was unclear if trainer clearly understood or misapplies module content<br><br>Some integration of module tools and target with participant's experience and needs		Consistently clear understanding and incorporation of module tools and targets throughout session<br><br>Frequent integration of module tools and target with participant's experience and needs		Prioritizing participant's needs and experiences and accurately integrating module tools and targets to address participant's needs	
